# lncRNA Rmst acts as an important mediator of BMP9-induced osteogenic differentiation of mesenchymal stem cells (MSCs) by antagonizing Notch-targeting microRNAs

**DOI:** 10.18632/aging.102583

**Published:** 2019-12-11

**Authors:** Zhicai Zhang, Jianxiang Liu, Zongyue Zeng, Jiaming Fan, Shifeng Huang, Linghuan Zhang, Bo Zhang, Xi Wang, Yixiao Feng, Zhenyu Ye, Ling Zhao, Daigui Cao, Lijuan Yang, Mikhail Pakvasa, Bin Liu, William Wagstaff, Xiaoxing Wu, Huaxiu Luo, Jing Zhang, Meng Zhang, Fang He, Yukun Mao, Huimin Ding, Yongtao Zhang, Changchun Niu, Rex C. Haydon, Hue H. Luu, Michael J. Lee, Jennifer Moriatis Wolf, Zengwu Shao, Tong-Chuan He

**Affiliations:** 1 Department of Orthopaedics, Union Hospital, Tongji Medical College, Huazhong University of Science and Technology, Wuhan 430022, China; 2 Molecular Oncology Laboratory, Department of Orthopaedic Surgery and Rehabilitation Medicine, The University of Chicago Medical Center, Chicago, IL 60637, USA; 3 Ministry of Education Key Laboratory of Diagnostic Medicine and the School of Laboratory Medicine; and the Affiliated Hospitals of Chongqing Medical University, Chongqing 400016, China; 4 Key Laboratory of Orthopaedic Surgery of Gansu Province, and the Departments of Orthopaedic Surgery and Obstetrics and Gynecology, The First and Second Hospitals of Lanzhou University, Lanzhou 730030, China; 5 Department of General Surgery, The Second Affiliated Hospital of Soochow University, Suzhou 215004, China; 6 Departments of Orthopaedic Surgery and Laboratory Medicine, Chongqing General Hospital, Chongqing 400013, China; 7 School of Life Sciences, Southwest University, Chongqing 400715, China; 8 Department of Burn and Plastic Surgery, West China Hospital of Sichuan University, Chengdu 610041, China; 9 Department of Orthopaedic Surgery, The First Affiliated Hospital, Guangzhou University of Chinese Medicine, Guangzhou 510405, China; 10 Department of Orthopaedic Surgery, The Affiliated Zhongnan Hospital of Wuhan University, Wuhan 430072, China; 11 Department of Orthopaedic Surgery, BenQ Medical Center Affiliated with Nanjing Medical University, Nanjing 210000, China; 12 Department of Orthopaedic Surgery, The Affiliated Hospital of Qingdao University, Qingdao 266061, China

**Keywords:** mesenchymal stem cells, BMP9, long noncoding RNAs, lncRNA Rmst, miRNAs

## Abstract

Understanding the bone and musculoskeletal system is essential to maintain the health and quality of life of our aging society. Mesenchymal stem cells (MSCs) can undergo self-renewal and differentiate into multiple tissue types including bone. We demonstrated that BMP9 is the most potent osteogenic factors although molecular mechanism underlying BMP9 action is not fully understood. Long noncoding RNAs (lncRNAs) play important regulatory roles in many physiological and/or pathologic processes. Here, we investigated the role of lncRNA Rmst in BMP9-induced osteogenic differentiation of MSCs. We found that Rmst was induced by BMP9 through Smad signaling in MSCs. Rmst knockdown diminished BMP9-induced osteogenic, chondrogenic and adipogenic differentiation in vitro, and attenuated BMP9-induced ectopic bone formation. Silencing Rmst decreased the expression of Notch receptors and ligands. Bioinformatic analysis predicted Rmst could directly bind to eight Notch-targeting miRNAs, six of which were downregulated by BMP9. Silencing Rmst restored the expression of four microRNAs (miRNAs). Furthermore, an activating Notch mutant NICD1 effectively rescued the decreased ALP activity caused by Rmst silencing. Collectively, our results strongly suggest that the Rmst-miRNA-Notch regulatory axis may play an important role in mediating BMP9-induced osteogenic differentiation of MSCs.

## INTRODUCTION

Multipotent mesenchymal stem cells (MSCs) are able to self-renew and differentiate into different lineages, including osteocytes, chondrocytes, and adipocytes [1–6]. MSCs are attractive sources of progenitor cells in the field of stem cell biology and regenerative medicine [4, 7–10]. The sequential events of osteogenic differentiation of MSCs resemble the processes occurring during bone development [11]. Although many signaling pathways, such as Wnt and Notch, can regulate osteogenic differentiation [3, 12–21], bone morphogenetic proteins (BMPs) are the most potent osteogenic factors [22–24]. BMPs belong to the transforming growth factor β (TGF-β) superfamily [3, 22, 23, 25], and there are at least 15 different BMPs identified in humans and rodents [22, 23, 26]. By analyzing the 14 types of BMPs’ osteogenic activities, we found that BMP9 (also known as growth differentiation factor 2, or GDF2) is one of the most osteogenic BMPs in MCSs both *in vitro* and *in vivo* [22, 24, 27–30], which may be at least in part explained by the fact that BMP9 is resistant to naturally occurring antagonist noggin [31]. We further demonstrated that the TGF-β/BMP type I receptors activin receptor-like kinase 1 (ALK1) and ALK2 are critical to BMP9 osteogenic signaling in MSCs [32].

However, the exact molecular mechanisms through which BMP9 induces osteogenic differentiation of MSCs are not fully understood. Deep sequencing has revealed that on average over 80% of the human genome is transcribed into RNA, while only less than 2% of the human genome is transcribed into protein-coding mRNA, leaving most of the RNA transcripts as noncoding RNAs (ncRNAs) [33–38]. Increasing evidence indicates ncRNAs, including long noncoding RNAs (lncRNAs), play important regulatory functions in normal and/or pathologic cellular processes [34–43]. Knockdown of some lncRNAs in embryonic stem cells and somatic progenitor cells caused defective differentiation pathways [44–46]. It was shown that lncRNAs associated with chromatin-modifying complexes and transcription factors to maintain the stemness of pluripotent stem cells [44, 45]. In other cases, some lncRNAs were shown to act in *cis* to regulate gene expression during development [46–49]. Thus, abundant evidence has implicated lncRNAs in regulating stem cell differentiation.

LncRNA Rmst was originally identified as a marker for the developing dopaminergic neurons in mouse [50] and has been shown indispensable for neurogenesis [45, 46]. Recent studies indicate that a trans-spliced tsRMST inhibited human embryonic stem cell differentiation [51], and RMST has been also implicated possessing a tumor suppressor role in triple-negative breast cancers [52, 53]. Thus, the biological functions of lncRNA Rmst remains largely elusive.

In this study, we investigate the possible role of lncRNA Rmst in BMP9-induced osteogenic differentiation of MSCs. We find that Rmst is induced by BMP9 through the Smad signaling pathway. Silencing Rmst expression effectively diminishes BMP9-induced osteogenic, chondrogenic and adipogenic differentiation *in vitro*, and significantly attenuates BMP9-induced bone formation. Mechanistically, silencing Rmst expression in MSCs leads to a decreased expression of Notch receptors and ligands. Bioinformatic analysis reveals that Rmst may directly bind to eight Notch-targeting miRNAs, six of which are downregulated upon BMP9 stimulation. Silencing Rmst in MSCs restores and/or enhances the expression of four of the eight miRNAs. A constitutively active Notch signaling molecule NICD1effectively rescues the decreased osteogenic activity caused by Rmst silencing. Collectively, our findings strongly suggest that the lncRNA Rmst-miRNA-Notch regulatory axis may play an important role in mediating BMP9 osteogenic signaling in MSCs.

## RESULTS

### lncRNA Rmst is induced by BMP9 in the intermediate early stage of osteogenic differentiation of mesenchymal stem cells (MSCs)

**Figure 1:**
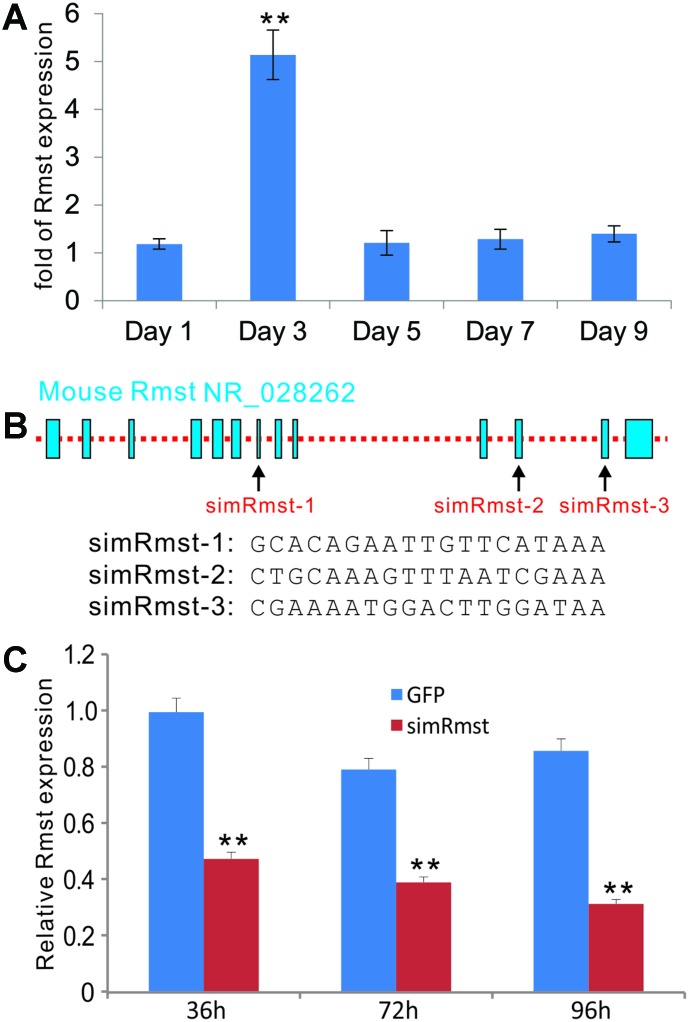
**BMP9-induced expression of lncRNA Rmst and construction of adenoviral vector-mediated siRNA knockdown of Rmst expression in MSCs.** (**A**) BMP9 induces the expression of lncRNA Rmst in MSCs. Subconfluent iMADs were infected with Ad-GFP or Ad-BMP9. At the indicated time points, total RNA was isolated and subjected to quantitative TqPCR analysis of Rmst expression. *Gapdh* was used as a reference gene. “**” p<0.001 when compared with Ad-GFP control group. Each assay condition was done in triplicate. (**B**) The transcriptomic arrangement of mouse lncRNA Rmst and the locations and sequences of three siRNA targeting sites are shown. (**C**) A recombinant adenoviral vector, called AdR-simRmst expressing the three siRNA sites, was constructed. To assess the Rmst knockdown efficiency, subconfluent iMADs were infected with AdR-simRmst or control Ad-GFP. At the indicated time point, total RNA was isolated and subjected to quantitative TqPCR analysis of Rmst expression. *Gapdh* was used as a reference gene. “**” p<0.001 when compared with Ad-GFP control group. Each assay condition was done in triplicate.

We first examined if BMP9 has any effect of Rmst expression in MSCs. We then infected the iMADs cells, an MSC line we previously characterized, with Ad-BMP9 or Ad-GFP control. Total RNA was collected at 1, 3, 5, 7, and 9 days post adenoviral infection and subjected to TqPCR analysis. We found that Rmst was significantly up-regulated at day 3 (Figure 1A), which represents the intermediate early stage of osteogenic differentiation. Furthermore, BMP9-induced Rmst expression was also observed in other MSC lines, including iMEFs and imBMSCs (data not shown).

We seek to determine whether Rmst plays an important role in BMP9-induced osteogenic differentiation. Based on the transcriptomic arrangement of mouse Rmst, we designed three siRNAs targeting the Rmst transcript (Figure 1B), and constructed the recombinant adenovirus AdR-simRmst. We further demonstrated that AdR-simRmst infected iMADs cells effectively and significantly suppressed endogenous Rmst expression in a time course-dependent fashion (Figure 1C).

### Silencing Rmst expression leads BMP9-induced expression of osteogenic, chondrogenic and adipogenic regulators and bone markers in MSCs

**Figure 2:**
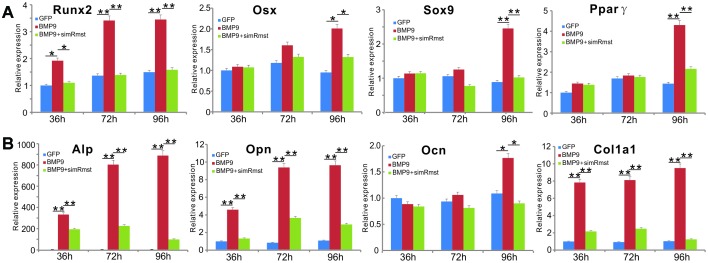
**Silencing lncRNA Rmst expression reduces BMP9-induced expression of osteogenic, chondrogenic and adipogenic regulators and bone markers in MSCs.** (**A**) Subconfluent iMADs were infected with Ad-BMP9 or Ad-GFP and AdR-simRmst. At the indicated time points, total RNA was isolated and subjected to TqPCR analysis with primers for mouse *Runx2, Sox9, Osx,* and *Pparγ*. *Gapdh* was used as a reference gene. “*” p<0.05 and “**” p<0.001 when compared with the Ad-GFP control group. Each assay condition was done in triplicate. (**B**) The cDNA samples prepared in (**A**) were further subjected to TqPCR analysis with primers for mouse *Alp, Opn, Ocn* and *Col1a1*. *Gapdh* was used as a reference gene. “*” p<0.05 and “**” p<0.001 when compared with the Ad-GFP control group. Each assay condition was done in triplicate.

As we previously showed that BMP9 can effectively induce tri-lineage (osteogenic, chondrogenic and adipogenic) differentiation in MSCs [22, 29, 30, 54], we tested whether silencing Rmst would impact the BMP9-induced expression of these lineage-specific regulators in MSCs. When iMADs cells were infected with Ad-BMP9, osteogenic regulator Runx2 expression was significantly up-regulated at 36, 72 and 96 hours after infection, while Runx2 downstream target Osx was up-regulated at 96h time point (Figure 2A). However, co-infection of AdR-simRmst effectively blunted BMP9-induced expression of both Runx2 and Osx (Figure 2A). Similarly, BMP9-induced expression of chondrogenic regulator Sox9 and adipogenic regulator Ppar*γ* was also effectively diminished by AdR-simRmst co-infection (Figure 2A), suggesting that Rmst may be an important mediator of BMP9-induced multiple-lineage differentiation of MSCs.

We also analyzed the effect of silencing Rmst on early and late osteogenic markers. When Rmst was silenced, BMP9-induced early marker Alp expression was significantly inhibited at the three tested time points (Figure 2B). Similarly, the BMP9-induced expression of later stage osteogenic markers Opn, Ocn and Col1a1 was significantly blunted by silencing Rmst expression in MSCs (Figure 2B). Interestingly, even though BMP9 up-regulated Rmst expression at day 3 (Figure 1A), silencing Rmst in iMADs cells seemingly inhibited BMP9-induced expression of early and late osteogenic markers at as early as 36h. One possible explanation of such phenomenon is that Rmst may have a high basal level of expression, which could be important for normal osteogenic differentiation of MSCs.

### Rmst silencing inhibits BMP9-induced ALP activity, matrix mineralization and adipogenic differentiation in MSCs

**Figure 3:**
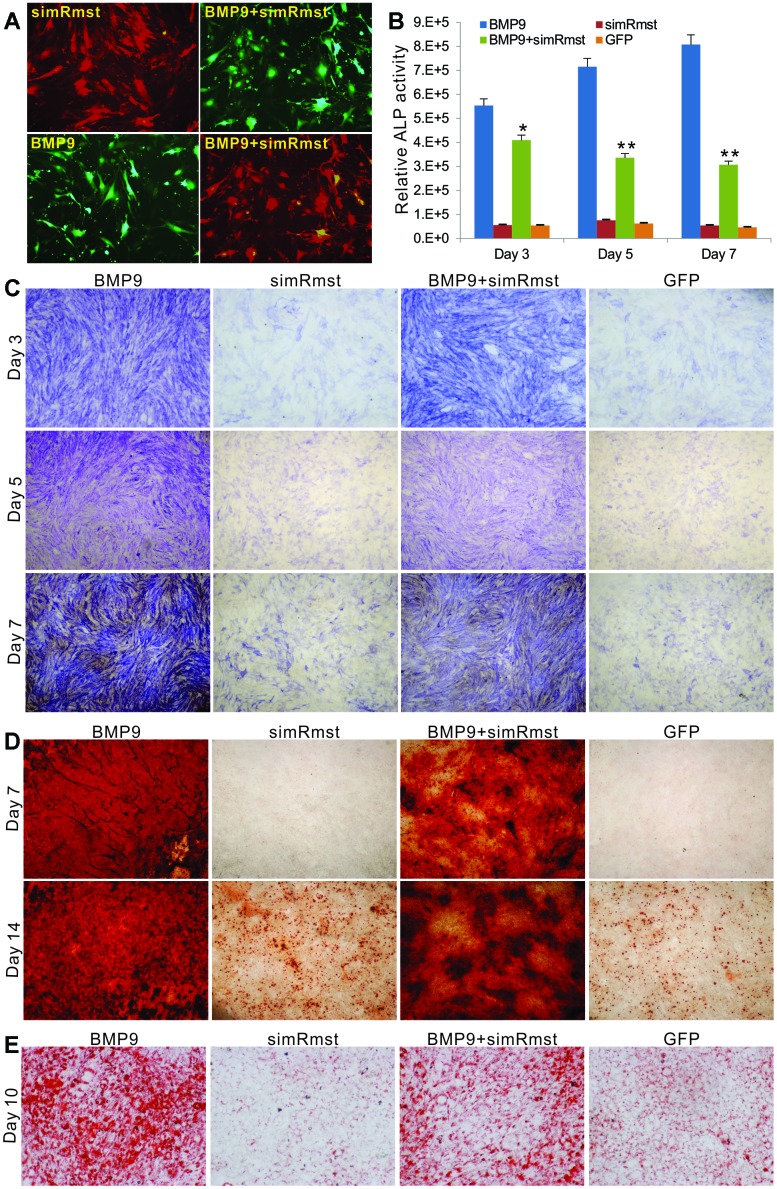
**Knockdown of Rmst diminishes BMP9-induced osteogenic and adipogenic differentiation of MSCs.** (**A**) AdR-simRmst was shown to infect the iMADs with high efficiency alone or co-infect with Ad-BMP9. Images were recorded at 48h post infection. Representative images are shown. (**B** and **C**) Downregulation of Rmst reduces BMP9-induced ALP activity in iMADs. Subconfluent iMADs were infected with Ad-BMP9, Ad-GFP, and/or AdR-simRmst. ALP activity was quantitatively determined at 3, 5 and 7 days after infection (**B**) or stained histochemically (**C**). Assays were done in triplicate. “*” p<0.05 and “**” p<0.001 when compared with the Ad-BMP9 alone group. Representative images are shown. (**D**) Silencing Rmst leads to reduced matrix mineralization induced by BMP9 in iMADs. Subconfluent iMADs were infected with Ad-BMP9, Ad-GFP, and/or AdR-simRmst, and cultured in mineralization medium. At day 7 and day 14, the infected cells were fixed and subjected to Alizarin Red S staining. Each assay condition was done in triplicate. Representative microscope images are shown. (**E**) Downregulation of Rmst reduces BMP9-induced adipogenesis in iMADs. Subconfluent iMADs were infected with Ad-BMP9, Ad-GFP, and/or Ad-simRmst. At 10 days post infection, the cells were fixed and subjected to Oil Red O staining. Each assay condition was done in triplicate. Representative microscopic images are shown.

We examined the effect of Rmst knockdown on BMP9-induced ALP activity, matrix mineralization and adipogenic differentiation of MSCs. The iMADs cells were effectively co-transduced by the adenoviral vectors, especially AdR-simRsmt and Ad-BMP9 (Figure 3A). Quantitative analysis indicated that BMP9-induced ALP activity was significantly blunted by Rmst knockdown in MSCs (Figure 3B). Similarly, the qualitative histochemical staining analysis demonstrated that BMP9-inudced ALP activity was effectively inhibited when co-infected with AdR-simRsmt at the three analyzed time points (Figure 3C). Moreover, we found that BMP9 induced robust matrix mineralization in MSCs, which was effectively inhibited when Rmst was silenced (Figure 3D).

We next tested whether BMP9-induced adipogenic differentiation would be affected by silencing Rmst in MSCs. As expected, BMP9 induced robust adipogenic differentiation as demonstrated by Oil-Red O staining assay (Figure 3E). However, silencing Rmst in the iMADs cells caused a significant decrease in BMP9-induced adipogenic differentiation (Figure 3E). Thus, these results indicate that Rmst may play an essential role in BMP9-induced multi-lineage differentiation of MSCs.

### Silencing Rmst expression attenuates the quantity and quality of BMP9-induced orthotopic bone formation *in vivo*

**Figure 4:**
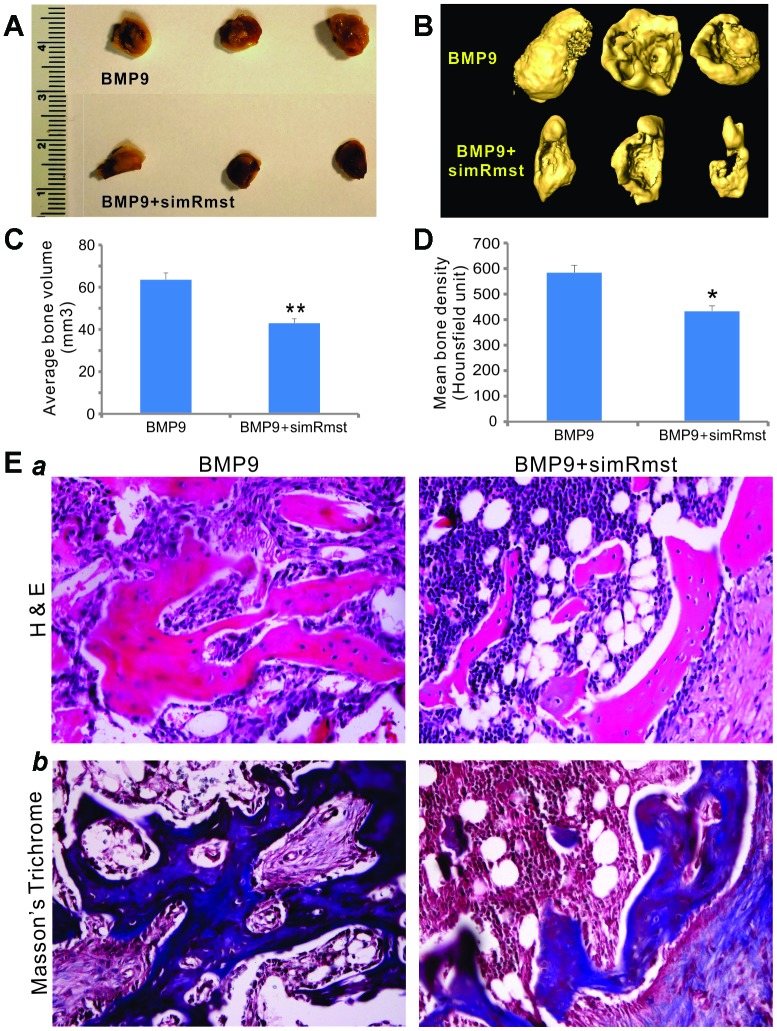
**Silencing Rmst expression attenuates BMP9-induced ectopic bone formation.** Subconfluent iMADs were infected with Ad-BMP9, Ad-GFP, and/or AdR-simRmst for 30h and collected for subcutaneous injection into the flanks of athymic nude mice. At 4 weeks after implantation, the mice were sacrificed and ectopic bone masses were retrieved. Representative macrographic images (**A**) and micro-CT isosurface images (**B**) are shown. No retrievable masses were found in the Ad-GFP or AdR-simRsmt alone group. The average bone volume (**C**) and mean bone density (**D**) were determined by analyzing micro-CT data using the Amira program. “*” p<0.05 and “**” p<0.001 Ad-BMP9 group *vs.* Ad-BMP9+AdR-simRmst group. (**E**) Histologic evaluation and trichrome staining. The retrieved masses were processed and subjected to hematoxylin and eosin staining (*a*) and Masson’s trichrome staining (*b*). Representative images are shown.

We determined whether Rmst knockdown in MSCs would impact BMP9-induced bone formation *in vivo*. When the iMADs cells were co-infected with Ad-BMP9, Ad-GFP, and/or AdR-simRstm and collected for subcutaneous injection into the flanks of athymic nude mice. Bony masses were successfully retrieved from both Ad-BMP9 and Ad-BMP9+AdR-simRmst groups at 4 weeks after implantation (Figure 4A) although no masses were detected in the Ad-GFP or AdR-simRmst group (data not shown). However, microCT analysis indicated that the average bone volume and mean bone density were significantly lower in the Ad-BMP9+AdR-simRmst group, compared with that of the Ad-BMP9 group (Figures 4B–4D).

Histologic evaluation further indicated that while BMP9 induced the formation of a robust trabecular bone network, silencing Rmst expression significantly decreased BMP9-induced trabecular bone formation (Figure 4E *panel a*). Trichrome staining also showed that BMP9-induced mature, well-mineralized bone matrix was significantly diminished when Rmst expression was silenced (Figure 4E *panel b*). Taken together, the above *in vivo* results strongly suggest that Rmst may play an important role in mediating BMP9-induced osteogenic differentiation of MSCs.

### BMP9 regulates the expression of Rmst through Smad signaling

**Figure 5:**
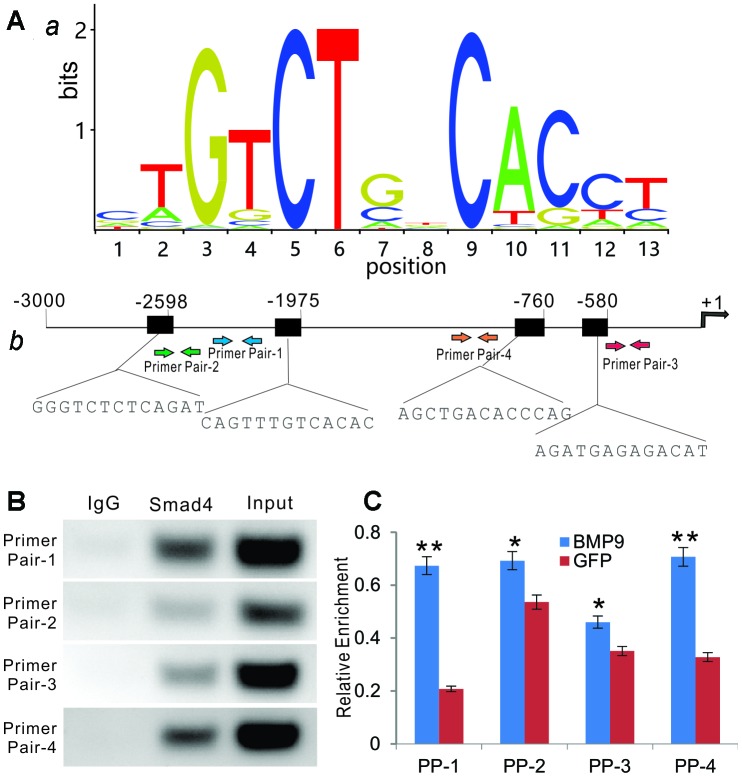
**BMP9 regulates Rmst expression through Smad signaling pathway.** (**A**) Bioinformatic prediction of putative Smad4 binding motif sequences using JASPAR. The representative position weight matrix for motif enriched in Smad4 binding sites by Chip-seq database (*a*). The sequences of putative binding sites and locations of PCR primer pairs are shown in (*b*). (**B**) ChIP analysis was performed with specific antibody for Smad4 in iMADs. Isotype matched IgG was used as a negative control. A whole cell extract (Input) was used as a positive control. (**C**) BMP9-induced binding of Smad4 to Rmst promoter. The iMADs were infected with Ad-BMP9 or Ad-GFP for 48h, and then subjected to anti-Smad4 ChIP pull-down as described in (**B**). RT-qPCR analysis was carried out to determine relative Smad4 promoter enrichment with different primer pairs. “*”, p<0.05, “**”, p<0.01, Ad-BMP9 group *vs.* Ad-GFP group.

To determine whether BMP9 regulates Rmst through the canonical Smad signaling pathway, we performed bioinformatic analysis of putative Smad4 binding motif sequences using JASPAR and identified representative position weight matrix for motif enriched in Smad4 binding sites in ChIP-seq database (Figure 5A, *panel a*). Four putative Smad4 binding sites were identified within the 3kb promoter region of mouse Rmst (Figure 5A, *panel b*).

We next performed ChIP analysis using anti-Smad4 antibody to pull down the Rmst promoter. In the semi-quantitative PCR analysis, we found that Primer Pair (PP)-1 and PP-4 enriched most by anti-Smad4 antibody, compared with that of the control IgG, while PP-2 and PP-3 also exhibited significantly weak but detectable signals (Figure 5B). To further test whether the Smad4 binding was BMP9-dependent, we infected iMADs with Ad-BMP9 or Ad-GFP and performed the anti-Smad4 ChIP assay as above, followed by quantitative PCR analysis to determine the enrichment of the detected four regions. We found that PP-1 and PP-4 fragments, to a much lesser extent PP-2, were significantly enriched upon BMP9 stimulation, while the proximal most PP-3 fragment was not enriched upon BMP9 stimulation (Figure 5C). Taken together, the ChIP assay results suggest that BMP9 may directly regulate Rmst expression through Smad signaling in MSCs.

### Rmst modulates Notch signaling pathway by neutralizing a panel of Notch-targeting miRNAs in BMP9-induced osteogenic differentiation

**Figure 6:**
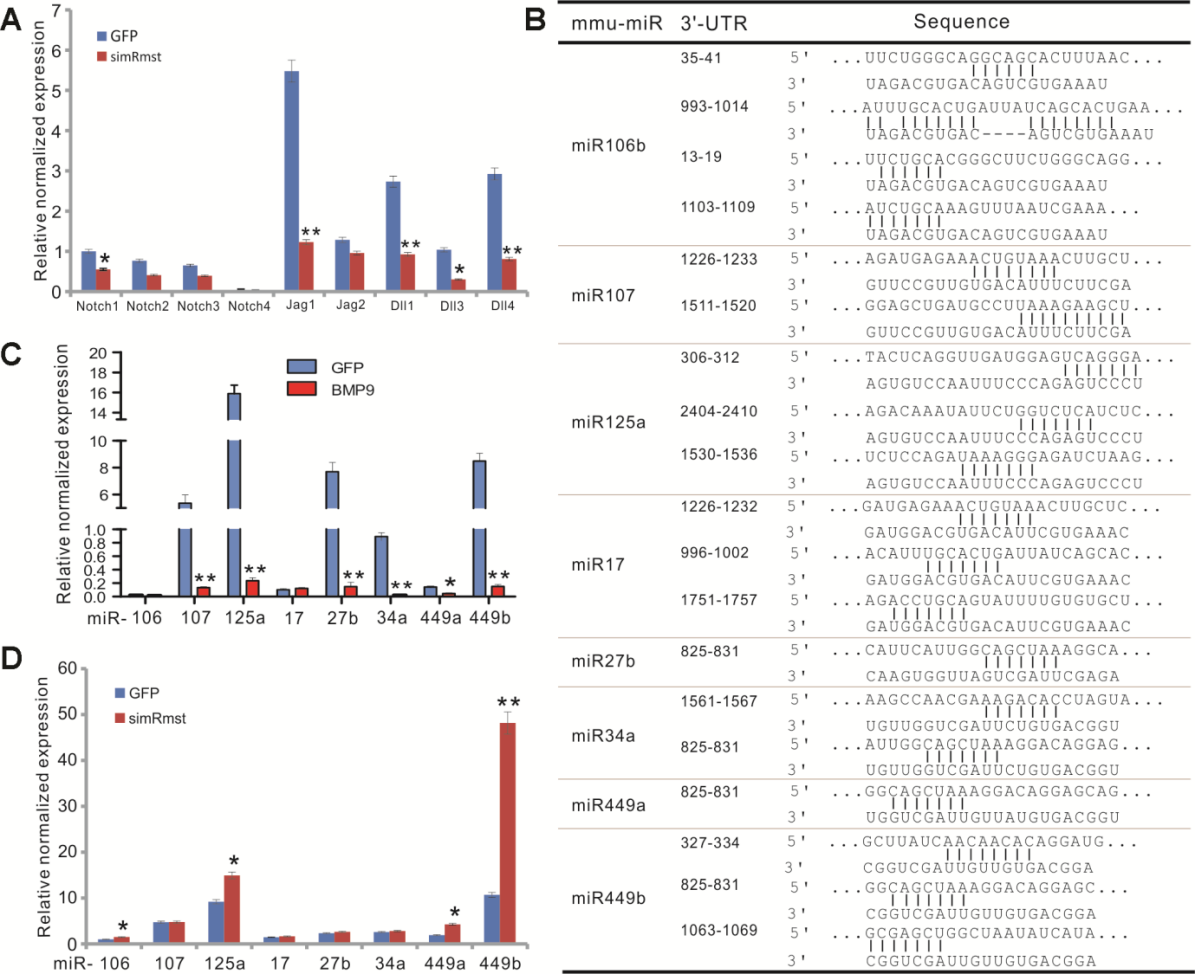
**Rmst modulates Notch signaling pathway by neutralizing a panel of Notch-targeting miRNAs in BMP9-induced osteogenic differentiation.** (**A**) Silencing Rmst reduces the expression of most Notch receptors and ligands. Exponentially growing iMADs were infected with Ad-GFP and Ad-simRmst for 72h. Total RNA was isolated and subjected to qPCR analysis use primers for the indicated genes. Each qPCR assay condition was done in triplicate. *Gapdh* was used as a reference gene. “*”, p<0.05, “**”, p<0.01, AdR-simRmst group *vs.* Ad-GFP group. (**B**) Putative target sites on Rmst for several Notch-targeting miRNAs. (**C**) BMP9 suppresses the expression of Notch-targeting miRNAs in MSCs. The iMADs were infected with Ad-GFP or Ad-BMP9 for 72h. Total RNA was isolated and subjected to TqPCR analysis. Each qPCR assay condition was done in triplicate. *Gapdh* was used as a reference gene. “*”, p<0.05, “**”, p<0.01, when Ad-BMP9 group *vs.* Ad-GFP group. (**D**) Silencing Rmst restores the expression of several Notch-targeting miRNAs in MSCs. The iMADs cells were infected with Ad-GFP or AdR-simRmst for 72h. Total RNA was isolated and subjected to TqPCR analysis. Each qPCR assay condition was done in triplicate. *Gapdh* was used as a reference gene. “*”, p<0.05, “**”, p<0.01, when AdR-simRmst group *vs.* Ad-GFP group.

We further investigated how Rmst would fulfill its regulatory role in mediating BMP9 signaling. One of the most important functions of lncRNAs is to modulate, or in most cases, to sponge miRNA functions, and we recently found that lncRNA H19 can sponge out microRNAs that normally target Notch receptors and/or ligands [55]. As Notch signaling plays an essential downstream role in mediating BMP9 osteogenic signaling [56], we first analyzed whether the expression of Notch receptors and/or ligands would be impacted by silencing Rmst expression; and found that silencing Rmst expression in MSCs led to a decreased expression of Notch1, Jag1, Dll1, Dll3, and Dll4 (Figure 6A).

Bioinformatic analysis indicates that Rmst may harbor multiple binding sites for eight Notch-targeting miRNAs (Figure 6B). To determine whether these miRNAs were functionally relevant, we analyzed the expression of the eight miRNAs in MSCs upon BMP9 stimulation, and found that six of them, miR-107, miR-125a, miR-27b, miR-34a, miR-449a, and miR-449b, were significantly suppressed upon BMP9 stimulation (Figure 6C).

To further confirm whether those miRNAs were functionally related to miRNAs, we analyzed the effect of the miRNA expression when Rmst expression was silenced. We found that silencing Rmst increased the expression of miR-106, miR-125a, miR-449a and miR-449b (Figure 6D), suggesting that these miRNAs may be directly sponged by Rmst.

**Figure 7:**
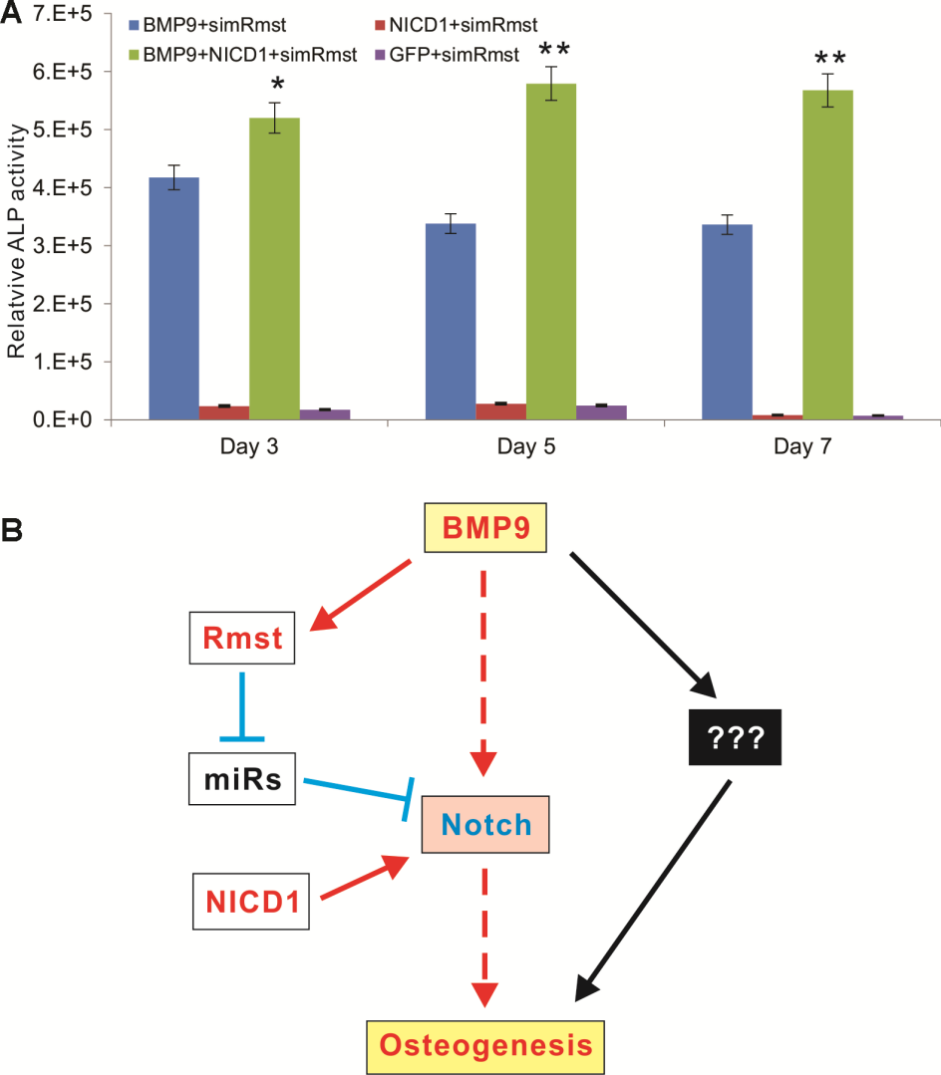
**A constitutive activation of Notch signaling rescues BMP9-induced ALP activity that is diminished by Rmst silencing.** (**A**) Subconfluent iMADs were co-infected with Ad-BMP9, Ad-GFP, Ad-simRmst, and/or Ad-NICD1. Quantitative measurement of relative ALP activity was determined at 3, 5 and 7 days after infection. Assays were done in triplicate. “*”, p<0.05, “**”, p<0.01, when Ad-BMP9+Ad-simRmst group *vs.* Ad-BMP9+Ad-NICD1+Ad-simRmst group. (**B**) A working model for the role of Rmst-miRNA-Notch regulatory loop in mediating BMP9-induced osteogenesis through Notch signaling. While BMP9 can induce osteogenic differentiation directly through Notch or other mediators, lncRNA Rmst provides an important delicate modulation of this process. The expression of Notch receptors and ligands is normally suppressed by a panel of miRNAs. BMP9 induces lncRNA Rmast, which subsequently sponges out those Notch-targeting miRNAs, leading to the de-suppression of Notch signaling and facilitating bone formation. The constitutive Notch activator NICD1 can bypass the Rmst-miRNA loop and directly activate Notch downstream events.

Lastly, we tested whether a constitutive activation of Notch signaling would rescue BMP9-induced osteogenic differentiation which was diminished by Rmst silencing. When the iMADs were co-infected with Ad-BMP9, Ad-GFP, Ad-simRmst, and/or Ad-NICD1, we found that exogenous expression of NICD1 effectively prevented the decrease in ALP activity caused by Rmst silencing; and in fact significantly increased ALP activity determined at 3, 5 and 7 days after infection (Figure 7A). Collectively, our results demonstrate that the Rmst-miRNA-Notch regulatory loop may play an important role in mediating BMP9-induced osteogenesis through Notch signaling.

## DISCUSSION

Originally identified in developing mouse liver [29, 57], BMP9 has been shown to play important roles in many cellular processes, including induction of osteogenic differentiation, maintenance of basal forebrain cholinergic neurons, inhibition of hepatic glucose production, regulation of lipid metabolism and iron metabolism, and modulation of angiogenesis [29]. Mechanistically, we identified several early downstream targets of BMP9 signaling, including the Notch downstream target Hey1 [29, 30, 58–63], and demonstrated BMP9 signaling extensively cross-talks with serval other signaling pathways, particularly Wnt and Notch signaling [30, 64–71]. Nonetheless, BMP9 is one of the least understood BMPs and thus many mechanistic aspects of BMP9 signaling remain to be fully understood.

Here, we investigated the role of lncRNA Rmst in BMP9-induced osteogenic differentiation of MSCs. We found that Rmst was induced by BMP9 at the intermediate early stage of osteogenic differentiation. Silencing Rmst effectively diminished BMP9-induced osteogenic, chondrogenic and adipogenic differentiation *in vitro*, and significantly attenuated the quantity and quality of BMP9-induced ectopic bone formation. ChIP analysis demonstrated that BMP9 induced Smad4 binding directly to the Rmst promoter region. Furthermore, we showed that silencing Rmst expression in MSCs led to a decreased expression of Notch1, Jag1, Dll1, Dll3, and Dll4. Bioinformatic analysis indicated that Rmst may directly bind to eight Notch-targeting miRNAs, six of which were downregulated upon BMP9 stimulation. Accordingly, the expression of four of the eight miRNAs can be restored or enhanced by silencing Rmst in MSCs; and that exogenous expression of NICD1 effectively rescued the decrease in ALP activity caused by Rmst silencing and in fact significantly increased ALP activity.

Based our findings, we propose a working model depicting that the Rmst-miRNA-Notch regulatory loop may play an important role in mediating BMP9-induced osteogenesis through Notch signaling (Figure 7B). While BMP9 can induce osteogenic differentiation directly through Notch or other mediators, lncRNA Rmst provides an important delicate modulation of this process. The expression of Notch receptors and Notch ligands is normally suppressed by a panel of miRNAs. BMP9 induces lncRNA Rmst, which subsequently sponges out those Notch-targeting miRNAs, leading to the de-suppression of Notch signaling and facilitating bone formation. The constitutive Notch activator NICD1 can bypass the Rmst-miRNA loop and directly activate Notch downstream events (Figure 7B).

Mounting evidence implicates ncRNAs in many physiological and/or pathologic processes, including osteogenic differentiation from MSCs [34–43]. We have recently investigated the role of lncRNA H19 in BMP9-induced osteogenic signaling [55]. Our results strongly suggest that the Notch signaling-associated miRNAs (e.g., miR-107, miR-27b, miR-106b, miR125a and miR17) may be modulated by H19 in response to BMP9 stimulation in MSCs [72–75]. Thus, our previous findings demonstrate that H19-miRNA-Notch regulatory loop may play an important role in mediating BMP9 osteogenic signaling in MSCs.

LncRNA Rmst was originally identified as a novel marker for mouse developing dopaminergic neurons, the dorsal midline cells of anterior neural tube, and the isthmic organizer [50]. It’s been recently shown that Rmst is indispensable for neurogenesis by binding to SOX2 promoter regions of neurogenic transcription factors, thus functioning as a transcriptional co-regulator of SOX2 [45, 46]. *RMST* orthologs, from human to frog, are highly conserved at their promoter regions, first exons, and splice sites [45, 46, 50, 76]. Silencing *RMST* in ReN-VM NSCs and H9-derived neural progenitors prevented neuronal differentiation [46], causing cells alternatively adopting a glia fate [45]. Conversely, *RMST* overexpression in human neural progenitors increased neuronal marker expression and a larger percentage of TUJ1-expressing neurons [46]. Interestingly, it was shown that RMST silencing protected against middle cerebral artery occlusion-induced ischemic stroke [77]. Another recent study reported that a trans-spliced tsRMST impeded human embryonic stem cell differentiation through WNT5A-mediated inhibition of the epithelial-to-mesenchymal transition (EMT) [51]. RMST has been also implicated in a tumor suppressor role in triple-negative breast cancers [52, 53]. It has been recently demonstrated that the dominant isoform of lncRNA Rmst is in circular RNA form [78]. Thus, our understanding about the biological functions of lncRNA Rmst is just a beginning.

In summary, we study the role of Rmst in BMP9 osteogenic signaling in MSCs. We demonstrate that Rmst is induced by BMP9 through Smad signaling at the intermediate early stage of osteogenic differentiation. Silencing Rmst expression effectively diminishes BMP9-induced osteogenic, chondrogenic and adipogenic differentiation *in vitro*, and significantly attenuates the quantity and quality of BMP9-induced ectopic bone formation. Mechanistically, silencing Rmst expression in MSCs leads to a decreased expression of Notch receptors and ligands. Bioinformatic analysis reveals that Rmst may directly bind to eight Notch-targeting miRNAs, six of which are downregulated upon BMP9 stimulation. Silencing Rmst in MSCs restores and/or enhances the expression of four of the eight miRNAs. A constitutively active Notch signaling molecule NICD1effectively rescues the decreased osteogenic activity caused by Rmst silencing. Collectively, our findings strongly suggest that the lncRNA Rmst-miRNA-Notch regulatory axis may serve as critical mediator of BMP9-induced osteogenic differentiation of MSCs.

## MATERIALS AND METHODS

### Cell culture and chemicals

HEK-293 cells were obtained from American Type Cell Collection (ATCC). HEK-293 derivatives 293pTP and RAPA cell lines overexpressing human Ad5 pTP and/or E1 genes were previously described [79, 80]. The conditionally immortalized mouse multipotent adipose-derived cells iMADs were previously described [81]. All cell lines were maintained in Dulbecco’s Modified Eagle Medium (DMEM) supplemented with 10% fetal bovine serum (Sigma-Aldrich, St Louis, MO, USA), containing 100 U/ml penicillin and 100 mg/ml streptomycin at 37°C in 5% CO_2_ as described [82–86]. Unless indicated otherwise, all other chemicals were purchased from Sigma-Aldrich (St. Louis, MO, USA) or Thermo Fisher Scientific (Waltham, MA, USA).

### Construction of recombinant adenoviruses Ad-BMP9, Ad-GFP, Ad-simRmst and Ad-NICD1

Recombinant adenoviruses were generated using the AdEasy technology as described [87–89]. The Ad-BMP9 was previously described [54, 56, 62, 69, 90, 91]. Briefly, the coding region of human BMP9 and the intracellular domain (NICD1) of human NOTCH1 were PCR amplified and subcloned into an adenoviral shuttle vector, and used to generate recombinant adenoviral vector, resulting in pAd-BMP9, pAdR-NICD1, which were subsequently used to generate recombinant adenoviruses in 293pTP or RAPA cells [55, 56, 70]. Ad-BMP9 also co-expresses enhanced green fluorescent protein (GFP), while AdR-NICD1 co-expresses monomeric red fluorescent protein (RFP). Ad-GFP was used as a mock virus control [92–94].

For the construction of siRNA expressing adenovirus, the three most optimal siRNA sites against mouse lncRNA Rmst were first selected using Dharmacon’s siDESIGN and/or Invitrogen’s BLOCK-iT RNAi Designer programs. The three siRNA cassettes were then constructed by Gibson Assembly into an adenoviral shuttle vector pAdTrace-OK, i.e., the three siRNA sites were engineered into the single vector, as described in our previously reports [95, 96]. The resultant shuttle vector pAdTrace-simRmst was used to recombine with the adenoviral backbone vector, resulting in pAdR-simRmst, which was subsequently used to generate recombinant adenovirus AdR-simRmst. AdR-simRmst virus co-expresses RFP as well. For all adenoviral infections, polybrene (8 μg/ml) was added to enhance infection efficiency as previously described [97].

### Total RNA isolation and touchdown quantitative Real-Time PCR (TqRCR) analysis

The cells were subjected to varied treatments. At the indicated time points, total RNA was isolated using the TRIZOL Reagent (Invitrogen, Carlsbad, CA, USA) according to the manufacturer’s instructions and subjected to reverse transcription reactions using hexamer and M-MuLV Reverse Transcriptase (New England Biolabs, Ipswich, MA, USA) as previously described [98–102]. The cDNA products were diluted 20- to 50-fold and used as PCR templates. The qPCR primers were designed by using the Primer3 Plus program [103]. The quantitative PCR analysis was carried out using our previously optimized TqPCR protocol [104]. Briefly, the 2x SYBR Green qPCR reactions (Bimake, Houston, TX) were set up according to manufacturer’s instructions. The cycling program was modified by incorporating 4 cycles of touchdown steps prior to the regular cycling program. *Gapdh* was used as a reference gene. All sample values were normalized to *Gapdh* expression by using the 2^-∆∆Ct^ method. The qPCR primer sequences are listed in Supplementary Table 1.

### Alkaline phosphatase (ALP) assays

ALP activities were assessed quantitatively with a modified assay using the Great Escape SEAP chemiluminescence assay kit (BD Clontech) and/or histochemical staining as described previously [67, 68, 105]. Briefly, osteogenic marker ALP activity was assessed at 3, 5, and 7 days after adenovirus infection. For the histochemical staining, the cells were fixed with 0.05% glutaraldehyde at room temperature for 10 min. After being washed with PBS, cells were stained with a mixture of 0.1 mg/mL of napthol AS-MX phosphate and 0.6 mg/mL of Fast Blue BB salt for 20 minutes. The stained cells were washed with PBS and recorded using a bright field microscope.

For the chemiluminescence assay, the cells were lysed by the Cell Culture Lysis Buffer (Promega, Madison, WI). Then 5μl of cell lysate, 5ul of substrate (BD Clontech) and 15μl of the Lupo Buffer were mixed well under a light-proof condition and incubated at room temperature for 20 minutes, followed by chemiluminescence reading. Each assay condition was performed in triplicate. The results were repeated in at least three independent experiments. ALP activities were normalized by total cellular protein concentrations among the samples.

### Matrix mineralization assay (Alizarin red S staining)

The iMADs cells were seeded in 24-well cell culture plates, infected with the indicated adenoviruses and cultured in the presence of ascorbic acid (50 mg/ml) and β-glycerophosphate (10mM). At the indicated time points, mineralized matrix nodules were stained for calcium precipitation by means of Alizarin Red S staining as described [31, 106–108]. Briefly, cells were fixed with 2.5% glutaraldehyde for 10 minutes. After being washed with PBS, cells were incubated with 2% Alizarin Red S at room temperature for 30 minutes, followed by washing with acidic PBS (pH 4.2). The staining of calcium mineral deposits was recorded under a bright field microscope. Each assay condition was performed in triplicate.

### Oil Red O staining assay

Exponentially growing cells were plated onto 24-well culture plates and infected with different adenoviruses. Oil Red O staining was performed at 10 days post-infection as described [54, 64, 91]. Briefly, cells were fixed with 10% formalin at room temperature for 10 min, followed by washing with PBS. The fixed cells were stained with freshly prepared Oil Red O solution (six parts saturated Oil Red O dye in isopropanol plus four parts water) for 60 minutes at room temperature, followed by washing with PBS. The staining of lipid droplets was recorded under a bright field microscope. Each assay condition was performed in triplicate.

### Subcutaneous stem cell implantation and ectopic bone formation

All animal use and care in this study followed the approved by the Institutional Animal Care and Use Committee (IACUP protocol #71108). All experimental procedures were carried out in accordance with the approved guidelines. Subcutaneous iMADs cell implantation procedure was performed as described [109–111]. Briefly, the iMADs cells were infected with different adenoviruses for 30h, the cells were harvested, resuspended in sterile PBS (80μl each injection), and injected subcutaneously into the flanks of athymic nude mice (Envigo/Harlan Research Laboratories; n=5/group, female, 5-6 week old; 2×10^6^ cells per injection site). The animals were maintained ad lib in the biosafety barrier facility. At 4 weeks after implantation, the mice were euthanized, and the implantation sites were retrieved for μCT imaging and histologic evaluation.

### Micro-computed tomographic (μCT) analysis

The retrieved specimens were fixed in 10% formalin and imaged using the micro-CT (μCT) component of the GE triumph (GE Healthcare, Piscataway, NJ, USA) trimodality preclinical imaging system. All image data were analyzed by Amira 5.3 (Visage Imaging, Inc.), and 3-D volumetric data and bone density were determined as previously described [62, 66, 112].

### H & E staining and Masson's trichrome staining

After being imaged, the retrieved tissues were fixed with 10% buffered formalin, decalcified and embedded in paraffin. Serial sections at 5μm of embedded specimens were carried out, and mounted onto treated slides. Then the sections of the embedded specimens were stained with hematoxylin and eosin (H & E). H & E staining and Masson’s trichrome staining were done as described [69, 81].

### Chromatin immunoprecipitation (ChIP) analysis

ChIP assay was carried out as previously described [61, 64, 113]. Approximately 5×10^6^ cells were used for each ChIP assay, and each assay condition was done in duplicate. Briefly, the iMADs cells were infected with Ad-BMP9 or Ad-GFP for 36h. The cells were crosslinked with 1% formaldehyde for 10 minutes and quenched by 125mM glycine (final concentration). Cells were lyzed and collected in lysis buffer (50mM HEPES/KOH, pH7.5; 1mM EDTA; 150mM NaCl; 1% Triton X-100; 0.1% SDS; 0.1% sodium deoxycholate) containing proteinase inhibitors (Roche, Indianapolis, IN), and subjected to sonication to shear genomic DNA into 500-1,000bp fragments. The sonicated lysate was centrifuged at 15,000x g at 4°C for 10 minutes to remove insoluble debris. One-third of the lysate was incubated with 5M NaCl at 65°C to reverse the cross-linking, followed by phenol-chloroform extraction and ethanol precipitation, and kept at -80°C as an input control for PCR analysis. The remaining two-thirds of the lysate were subjected to immunoprecipitation using Smad4 antibody (Santa Cruz Biotechnology) or mouse IgG at 4°C overnight, and then incubated with Protein G beads for 4 hours at room temperature. Immuno-precipitants were sequentially washed with lysis buffer twice, followed by washing once with wash buffer (lysis buffer with 0.5 M NaCl). After the final wash, 200μl of elution buffer (50 mM Tris-HCl, 10 mM EDTA, pH 7.5, 1% SDS) was added and rotated at room temperature for 15 min to elute the protein/DNA complexes. NaCl (5 M) was added to the recovered eluent mix and incubated at 65°C for 4h to reverse the formaldehyde cross-linking, incubated with RNase A for 30 minutes at 37°C, and Proteinase K for 1h at 45°C. The DNA was extracted with phenol-chloroform, ethanol precipitated, and resuspended in double-distilled water for semi-quantitative PCR analysis and TqPCR analysis.

### Statistical analysis

All quantitative studies were carried out in triplicate and/or performed in three independent batches. Microsoft Excel program (Redmond, WA, USA) was carried out to calculate standard deviation (S.D.). Statistically significant differences between samples were determined by one-way analysis of variance. A value of p<0.05 was considered statistically significant when one comparison was being made.

## Supplementary Material

Supplementary Table 1
