# Supplementary material for: lncRNA Rmst acts as an important mediator of BMP9-induced osteogenic differentiation of mesenchymal stem cells (MSCs) by antagonizing Notch-targeting microRNAs
Source: Aging (Albany NY). 2019 Dec 11;11(24):12476–96. doi: 10.18632/aging.102583 (PMC6949095; doi:10.18632/aging.102583)
Supplement: Supplementary Table 1 [file aging-11-102583-s001..pdf]

SUPPLEMENTARY TABLE

Supplementary Table 1. List of qPCR Primers.

Genes/Transcript	qPCR Primer sequences		Accession Number
	Forward	Reverse	
mouse Rmst	CCACAGAGTCGGCTGCAA	TTCACCGGCAAGGCAGAG	NR_028262
mouse Opn	CCTCCCGGTGAAAGTGAC	CTGTGGCGCAAGGAGATT	NM_001204201.1
mouse Ocn	CCTTCATGTCCAAGCAGGA	GGCGGTCTTCAAGCCATAC	NM_001032298.3
mouse Runx2	CCGGTCTCCTTCCAGGAT	GGGAACTGCTGTGGCTTC	NM_001146038
mouse Osx	GGGAGCAGAGTGCCAAGA	TACTCCTGGCGCATAGGG	NM_130458.3
mouse Alp	CCCCATGTGATGGCGTAT	CGGTAGGGAGAGCACAGC	NM_001287172.1
mouse Col1a1	GAGCGGAGAGTACTGGATCG	GCTTCTTTTCCTTGGGGTTC	NM_007742.3
mouse Sox9	CACCTGTGCCTCTCAGAACA	TGAGGAAAGCTCCAACAACC	NM_011448.4
mouse Pparγ	GAAGCCGTGCAAGAGATCA	ATGAATCCTTGGCCCTCTG	NM_011146.3
mouse Gapdh	ACCCAGAAGACTGTGGATGG	CACATTGGGGGTAGGAACAC	NM_008084.3
mouse Notch1	CCCGCATTCCAACATCTC	GGTCCTGCATCCCACATC	NM_008714.3
mouse Notch2	AGCAGGAGGGGCAGGTAG	GGTTCGCTCAGCAGCATT	NM_010928.2
mouse Notch3	CTGGCTCCAGATGCCTGT	GGGGACAGCACCTCACAC	NM_008716.2
mouse Notch4	CCGTCCTGGTTTCACAGG	GACTTCCGTCAGGGCAGA	NM_010929.2
mouse Jagged1	CCAACACGGTCCCCATTA	TTGGCAAAGCGGACTTTC	NM_013822.5
mouse Jagged2	CACGCTGGCATGATCAAC	TGTTGCAGGTGGCACTGT	NM_010588.2
mouse Dll1	CCGGTTTGTGTGTGACGA	CCAGGGTCGCACATCTTC	NM_007865.3
mouse Dll3	GGGCTTCGATGTGAGGTG	GAAACCAGGTGGGCAATG	NM_007866.2
mouse Dll4	GGGCCTTCCTTCTGCATT	ACTCTTGGCGGGTTCACA	NM_019454.3
mmu-miR-106b	CCTGCTGGGACTAAAGTGCT	TACCCACAGTGCGGTAGC	NR_029658.1
mmu-miR-107	TCAGCTTCTTTACAGTGTTGCC	AGCCCTGTACAATGCTGCT	NR_029783.1
mmu-miR-125a	CCCTTTAACCTGTGAGGACGT	GGCTCCCAAGAACCTCACC	NR_029539.1
mmu-miR-17	CAAAGTGCTTACAGTGCAGGT	GTGCCCTCACTGCAGTAGA	NR_029785.1
mmu-miR-27b	AGGTGCAGAGCTTAGCTGA	GCCACTGTGAACAAAGCGG	NR_029531.1
mmu-miR-34a	TGGCAGTGTCTTAGCTGGT	CAATGTGCAGCACTTCTAGGG	NR_029751.1
mmu-miR-449a	TGTGATGGCTTGGCAGTGT	TTAGCTGGTGCCGCTCAC	NR_029961.1
mmu-miR-449b	AGACTCGGGTAGGCAGTGT	GTGGCAGGGTAGCTGTGG	NR_030602.1
